# Quo Modo?

**Published:** 1856-03

**Authors:** 


### QUO MODO?

*In what manner?* That is the translation. We must use Latin, Greek, French, German, Spanish, Italian, Hebrew—all of them, or that astute entity, the people, will conclude we are know nothings. To be sure, it is not a particular indication of learning, or of good taste either; in fact, it is a waste of space, ink, type and paper, for the words of these languages must be interpreted into our own, else we might as well have written in modern Chinese or ancient Chaldaic. But then we can tickle the people at the expense only of looking into our Dictionary (polyglott) of Quotations, take the first phrase that looks learned, master its definition, and then write up to it. Besides, we have a good memory for definitions, if not in name, yet in idea; as good as the boy who never could pronounce the first letter of the alphabet. "I know him very well by sight, sir, but rot me if I can call his name," was the steady excuse of little stupid for not calling "A."

We once learned the definition of *sine qua non*, and never have forgotten it. There is a large class of Anti-Know-Nothings: one of these, who never plead guilty of ignorance of any thing, was listening to a brother or first cousin of his, who never lost an opportunity of putting himself forward, reading an important item of news to a crowd around a country post-office door. The reader bolted through the phrase with tolerable courage and success, but had scarcely cleared the bridge, when one of the crowd exclaimed, "What's that? I never heard tell of such a sign as that." "O, go on," said know-all, "it's an island in Passamaquoddy Bay; I've seen it many a time!"

Well, as we were saying "*In what manner?*" of course we must *make the connection* between what we say, and health, or it will be flying the track, and the race for fame and fortune, especially the latter, will be lost for aye.

*In what manner*, then, is a few barrels of molasses and water turned into a two hundred thousand dollar dwelling in Fifth Avenue, besides having a spare thousand or two, every once in a while, to help build a church and to aid in the temperance cause—religion and liquor temperance being the most popular of all causes, temperance in food and the passions, having no friends, is simply laughed at. Surely we will come to a focus by-and-bye, as soon as we describe "*in what manner*" a Broadway hotel has been built and furnished, at a cost of a quarter of a million of dollars, out of a cargo of aloes. Well, the aloes, bitter as gall though they are, were eaten morning, noon, and night—eaten by "the people" year after year, until of divers cargoes not a single ounce is left; not only eaten willingly, gladly, but at a premium, a cash premium, of above twenty-five cents a spoonful.

"*In what manner*, I would like to know," inquires the reader —*In what manner?* QUO MODO? were "the people" induced to do such an unaccountable thing?

Why, a country editor was persuaded to say, for a consideration, for of course you could not expect an editor to talk, or write, or print for nothing, and thereupon a hundred city editors were hired to repeat, as coming from this country editor, for a proportional consideration, say as one to one thousand, the following (names suppressed)

[Advertisement.]
*From the —— Herald.*

CLEANSE THE SYSTEM.—At this season of the year a general purification of the human system is vitally important. During the Winter season, a vast amount of impurity gathers in the system, producing humors and lassitude, and eventuating in disease if not removed; and as nature provides for the external purification of her dominions at this season by electricity, shower and storm, so man should watch over his own system and purify it. For this purpose we are persuaded that ——— Pills stand pre-eminent. For twenty years past they have been in general use throughout this country, and at the present time there is not another so popular medicine for purifying the system in use; besides which, they are cheap, and pleasant to take.

Now it so happens that this advertisement expresses the truth in every line, with one exception, especially as to the "pre-eminency of the pills," for they literally stand six stories high above ground, and two below, and to progress indefinitely deeper hereafter, pills, patentee, and all.

Although this advertisement may be strictly and literally true, yet a false use is made of it,—a use which is ruinous to the health and life of multitudes, thus:

The certain tendency of the use of aloes, is to leave the bowels more and more costive, requiring a freer and freer employment of it, ending in piles, or fistula, attended with consequences painful, horrible, disgusting.

That "*a vast amount of impurities gather in the system during the winter*," is a concentrated untruth; the advertiser would have saved his reputation, had he expressed no opinion as to the time during which the impurities had gathered, and simply stated the general fact, that they were more or less present in all, in spring. The system ought to be purified, and the pills are pre-eminently popular, and they do aid in purifying the system, but the ultimate, certain, inevitable results,—look at them! Arsenic eaten *secundem artem*, brings plumpness to the cheeks and roses to the skin and sparkling to the eye, and in the end brings death!

In our article in the June number, "A Life-Saving Thought," we explained that the blood does become impure in the Spring, because of our eating with the appetite of winter, when we do not need half as much food, in consequence of our exercising less vigorously, and also because we need less warmth in summer than in winter.

If we would eat but half as much in early spring, the blood, which bracing winter bequeaths in perfect purity to spring, would remain pure; or, if in default of this precaution, when we find ourselves ill we would diminish our food within proper bounds *forthwith*, and take large amounts of daily pleasurable exercise in the open air, not involving fatigue, the blood would purify itself in nature's own safe, harmless and beneficent way, just as certain as a clear running spring will purify itself after disturbance, if it is only let alone.

But what a bootless work it is to write thus! Not one in a score of all who read this article, will make a practical and rational use of it, but when spring comes, will continue to eat on, with a winter's appetite, until the system is so charged with a thick, loaded, imperfect blood, that nothing can save the body from impending disease, but eating less food or by swallowing more pills, the latter being preferred by most persons, and they the very ones who do not call in a physician for fear he would give them medicine, and yet they turn right round and take ten times as much ignorantly, and on their own responsibility, as an educated physician would have given them. Those who take medicine on their own responsibility, are precisely those who are "*all the time taking something;*" the reason of this is two-fold.

1st. Patent medicines are temporary in their effects.

2d. They alleviate or smother, instead of eradicating disease.

A patent pill, or balsam, or bitters, is taken for a specified symptom; relief follows; this gives confidence in its efficacy, which is forthwith extended to some other symptom; as it "helped" a cough, it may help a colic; so, very soon it is announced by the sanguine recipient, it is good for almost anything, and consequently it soon becomes a cure-all, and for every trifling little ailment the favorite remedy is resorted to, while all this time the person forgets two or three very observable facts.

1st. That somehow or other he is more frequently " ailing " than he used to be.

2d. That larger doses of his favorite remedy have to be taken.

3d. That new ailments are appearing.

To illustrate. A gentleman recently informed me that —— Pills were taken by his grandmother with apparent advantage some years ago, and that the habit had grown on her to such an extent that now she was compelled to take them every few weeks; and that whereas at first, one or two were sufficient to give relief, now "*a level table-spoonful*" were needed at a single dose, in order to secure a decided result.

It is known to many, that " Morison's Pills " had a great popularity at one time in England, until it was charged in court that they had killed a patient. The inventor himself was probably sincere in his belief of their sovereign and universal efficacy, for on one occasion he became sick, and having taken his pills several days without encouraging results, his friends insisted on his using some other medicine. This he resolutely declined to do, saying, that if his pills did not cure him nothing else could, so in a single day he took upwards of one hundred and eighty, and——died!

Rhubarb and aloes are large constituents of almost every patent pill of wide popularity, for moving the bowels. The ultimate effect of either, is to leave the system in a more constipated condition; *they cannot be taken without such a result*, and constipation is the fruitful cause of piles, falling of the lower bowel, and fistula, a disease, which for months, and sometimes for years before death, leaves the patient in that most pitiable and disgusting condition, wherein the excrements pass without the will, the urine dribbles away incessantly, and life is a foul and ceaseless torture! So habitual patent pill-takers have in prospect "*a time of it*" above ground, not less impressive than the knavish makers of the same have below.

---

TO TAKE INK OUT OF LINEN.—Saturate the spots with melted tallow, then wash in suds.

